# Editorial Perspective: Prioritizing child and adolescent mental health research in the context of war

**DOI:** 10.1111/camh.70002

**Published:** 2025-06-24

**Authors:** Alexa X.D. Zhang, Olga Osokina, Sanju Silwal, Norbert Skokauskas, Andre Sourander, Matthew Hodes

**Affiliations:** ^1^ Central and North West London NHS Foundation Trust London UK; ^2^ Addiction Psychiatry and Medical Psychology Donetsk National Medical University Kropyvnitskyi Ukraine; ^3^ Psychiatry and Physical Rehabilitation Kyiv Medical University Kyiv Ukraine; ^4^ Research Centre for Child Psychiatry, INVEST Flagship University of Turku Turku Finland; ^5^ Regional Centre for Child and Youth Mental Health and Child Welfare, IPH Norwegian University of Science and Technology Trondheim Norway; ^6^ Department of Child Psychiatry University of Turku and Turku University Hospital Turku Finland; ^7^ Division of Psychiatry Imperial College London London UK

**Keywords:** Child and adolescent psychiatry, child and adolescent mental health, war, ethics, child and adolescent psychiatry research during wars, refugee

## Abstract

Given the high level of exposure of children and adolescents (CA) to war and associated adversities (1 in 6 children live in war zones at the time of writing) and very detrimental effects on their mental health, we argue for the need to involve them in mental health research. Although there is abundant literature on CA mental health research in nonwar and postwar settings, the findings cannot be directly extrapolated to war contexts. Viewing CA as too vulnerable, as well as ethical and methodological challenges are among the reasons for the low level of research on this topic. Drawing on the available literature and our experience conducting epidemiological studies on the impact of the war following the Russian invasion of Ukraine, we highlight the complexity and importance of conducting CA mental health research during wars and the key ethical and methodological considerations. We advocate the active inclusion of CA as research participants and beneficiaries as a first step to building an evidence base for treatment and services.

## Introduction: Why this topic is important?

We face a paradox that children and adolescents (CA) are one of the most vulnerable groups during war, yet their subjective mental and emotional experiences and needs are often excluded from empirical studies in the war context, further excluding them from research benefits. We discuss several ethical and methodological issues that might have resulted in CA being excluded and propose that their inclusion during war should be prioritized with appropriate research adaptations.

One reason for investigating CA in war contexts is the sheer number affected. More than 468 million children (1 in 6) globally lived in war zones in 2023, and about 250 million were living in the most lethal wars (Save the Children, n.d.). The destructiveness of war and organized violence has led to high levels of deaths, and amongst survivors' distress and often mental health problems including posttraumatic stress disorder, anxiety disorders, depression, and suicidality that may last months or years and are associated with significant social impairment. This is a vitally important yet neglected area for research.

Although there is an abundant literature on CA mental health research in nonwar and postwar settings, the findings may not be generalizable to CA living in war zones. Informants often provide inadequate information during wars, and CA need to have their own voice to communicate their experiences first‐hand, such as their fears and wish to regain a sense of safety. CA's subjective experiences are often not reliably known or reported by adult caregivers (Van Roy, Groholt, Heyerdahl, & Clench‐Aas, 2010). For instance, CA may not disclose shameful war experiences such as sexual assault and underreport symptoms because of fear of heightening caregivers' distress. While caregivers, of course, may be aware of visible distress (e.g., crying, clinging, withdrawal, insomnia, loss of appetite, nocturnal enuresis), they also underreport emotional symptoms in nonwar settings (Van Roy et al., 2010). Therefore, CA need to report symptoms themselves, including their fears, nightmares, level of avoidance, hyperarousal, concentration difficulties, and suicidal thoughts that frequently occur during wars.

CA already marginalized in their communities may face further stigmatization if not actively included to understand their needs. Examples of these groups include the very young and female adolescents, pregnant adolescents, those with physical or mental disabilities, adolescents separated from their families, adolescent heads of households, adolescent survivors of sexual‐/gender‐based violence, adolescent girls selling sex, and children associated with armed forces. Despite their vulnerabilities, these CA can benefit from sensitively planned research. Postwar studies exemplify this, such as the Longitudinal Study of War‐Affected Youth in Sierra Leone from 1991 to 2002, which offered novel perspectives on risk and protective factors in community reintegration, social relationships, and psychosocial function in former child soldiers (Betancourt, Keegan, Farrar, & Brennan, 2020). There is significantly less such investigation of CA during wars than in postconflict situations. Reasons for this include practical problems conducting research during war, most crucially the risk to investigators and participants and prioritizing physical and material needs. An example is the war in Gaza in the months following the Hamas‐led attack in Israel on October 7, 2023. However, there may be other reasons limiting the prevalence of CA mental health research in wars. We discuss some of these barriers below.

### Perceived categorical vulnerability leading to exclusion from research

CA are inherently prone to perceived categorical vulnerability during research due to age. However, CA are heterogeneous in terms of age and maturity, with different physical, mental, and emotional capacities, as well as socioeconomic profiles, which means they are positioned differently in terms of risks and vulnerabilities created by conflicts. An attempt to “protect” CA from research also excludes them from the consequential benefits. Instead of seeing vulnerability as a static factor secondary to group characteristics, it should be considered alongside other contextual factors. Certain subgroups of children may be unsuitable for research in one context using one set of research methodologies but not in another. Where inclusion is necessary to understand the needs of a specific subgroup, adequate safeguards and all risk minimization measures should be implemented.

### Ethical considerations in researching children and adolescents' mental health during war

Postconflict studies in refugee camps may provide valuable lessons that can be generalized to ongoing conflict situations, but this approach does not usually answer key questions regarding the mental health of CA living in war contexts. Much of the existing research has been cross‐sectional and primarily focuses on assessing CA psychopathology in relation to past conflict exposure. The scarcity of prospective longitudinal studies, especially ones that incorporate developmental and ecological perspectives, has resulted in a lack understanding of how specific risk and resilience factors shape psychopathology and development over time that could inform interventions (Betancourt, 2011). Retrospective approaches are associated with biases, such as recall bias, particularly prevalent in situations associated with high psychological arousal and mass displacement (Given‐Wilson, Hodes, & Herlihy, 2018). Documenting specific war events, including moves associated with displacement and associated distress may not be reliably reported long after they were experienced.

Carrying out research in war zones within the limitations of war‐damaged research infrastructure has many challenges, including barriers to building trust with deeply traumatized communities, difficulties in obtaining informed consent, and data collection. Psychiatric interviews may be regarded as stress inducing, but when conducted appropriately, can also act as a form of therapeutic intervention in an already traumatized population. For example, an interview‐based study with Bosnian refugee parents and adolescents showed that nearly all participants found the research process a positive experience (Dyregrov, Dyregrov, & Raundalen, 2000). It is therefore important to consider these factors when designing research and acknowledge the possibilities and limitations of research conducted in such environments.

Other risks that need to be considered include data security and risk to the researched community and researchers. Safety planning should be undertaken prior to all research in emergencies, but the challenge lies in the application of research protocols during wars, where access to treatment and services is severely constrained. Safety planning should specifically include the following: equipping researchers with child‐friendly and culturally sensitive research methods, tools to recognize and respond to distress, an awareness of how researchers' own characteristics affect the process, robust risk assessment, clear and fair selection criteria, referral protocols, adapted methods and a continuous assessment of consent. Ensuring a research design appropriate to the developmental stage and contexts of the participants makes it as participatory, noninvasive and stress‐free as possible.

Obtaining informed consent based on local and international standards is essential (Inter‐Agency Standing Committee (IASC), 2014). CA must understand the benefits and risks of research and the process of informed consent should be a dynamic process. A degree of flexibility in the way consent is obtained may be necessary to secure key components of consent during war contexts. Research during war should protect participants' rights to confidentiality. Researchers must also be aware of the limits of confidentiality – for example, when to breach confidentiality to protect participants. Research collaboration with institutions, such as the governments and humanitarian organizations, need to establish agreements and ensure ethical oversight and participant protection.

Whether payments could be incorporated ethically in war research depends on several factors. Where research is noninvasive and low risk, monetary payments or in‐kind gifts can encourage participation and reimburse participants for costs incurred. Incentives should be appropriate to the context. The type and amount of incentives should not apply undue pressure on stakeholders' abilities to make informed decisions around participation. Although material hardship is a reality associated with war and organized violence, a discussion about incentives with stakeholders can be beneficial in understanding complex motivations to participate and thereby attempting to make the participation process as equitable as possible.

### Methodological considerations for research during war

CA psychiatric population studies typically recruit participants using administrative systems (e.g., housing, school, primary care or census data). During wars, these systems can no longer be relied on to recruit representative samples due to population displacement. Alternative strategies such as recruitment through referrals from other organizations or support schemes, or targeting those in camps for the displaced, have been employed in postconflict settings.

Regarding the instruments and data collection, clear systems are needed for administration, collection, and safe storage of completed responses. Digital data collection methodologies offer rapid and safer communications and enable large groups to be recruited quickly. Our epidemiological study of adolescents in Ukraine included data collection in 2023–2024 following the full‐scale Russian invasion of 2022 using school rolls in the same areas as the first phase of data collection (data collected 2016–2017) (Osokina et al., 2023). It took place during the high level of bombing and destruction. The resulting displacement of populations meant that adolescents who were receiving schooling remotely were enrolled in the study through digital communication and completed electronic questionnaires.

However, there are logistical challenges that need to be addressed include ensuring data privacy and comprehensive digital access for participants. Given the current limited understanding of mental health issues in CA during war contexts, using mixed or qualitative methodologies may be informative. An example of methodological adaption to minimize stigmatization is the use of school observations and classroom‐based activities involving all pupils without singling out participants.

## Discussion

CA are often overlooked as participants in research especially in war contexts. We have argued for the need to hear their voices, both in quantitative and qualitative research, as carers or other informants cannot speak adequately for them, and often underreport their experiences. Research needs to be adapted in war contexts to achieve safety for participants and researchers. The expansion of digital applications may facilitate the execution of research. There have been calls for collaborative international research that links investigators in high income and low‐income countries but trust between partners is crucial given the sensitive nature of mental health research. In contexts of extreme high level of war, CA mental health research will not be feasible or appropriate, but in prolonged war as occurred in Ukraine since 2014, it may inform service planning and delivery.

## Conflict of interest statement

All authors reported no biomedical financial interests or potential conflicts of interest.

## Funding information

Dr. Sourander has received funding from the European Research Council (ERC) under the European Union's Horizon 2020 research and innovation program (grant agreement No. 101020767), and from the Research Council of Finland (decision number: 345546). Dr. Norbert Skokauskas received funding from The UNA (The Ukrainian, Norwegian, Armenian and the World Health Organization Regional Office for Europe Partnership) (2016–2019). The funders played no role in any aspect of the study or manuscript.

## Author contributions

MH, AS, SS, OO, and NS conceptualized this study. AXDZ supported the literature review and prepared the first draft. All authors revised the manuscript for important intellectual content and provided final approval of the version to be published.

## Ethics statement

Not applicable.

## Data Availability

Not applicable.
